# Mechanistic insights on the mode of action of an antiproliferative thiosemicarbazone-nickel complex revealed by an integrated chemogenomic profiling study

**DOI:** 10.1038/s41598-020-67439-y

**Published:** 2020-06-29

**Authors:** Enrico Baruffini, Roberta Ruotolo, Franco Bisceglie, Serena Montalbano, Simone Ottonello, Giorgio Pelosi, Annamaria Buschini, Tiziana Lodi

**Affiliations:** Department of Chemistry, Life Sciences and Environmental Sustainability, University of Parma, Parco Area Delle Scienze, 11/A, 43124 Parma, Italy

**Keywords:** Genomics, Chemical genetics, Mechanism of action, Metabolic pathways, Functional genomics

## Abstract

Thiosemicarbazones (TSC) and their metal complexes display diverse biological activities and are active against multiple pathological conditions ranging from microbial infections to abnormal cell proliferation. Ribonucleotide reductase (RNR) is considered one of the main targets of TSCs, yet, the existence of additional targets, differently responsible for the multifaceted activities of TSCs and their metal complexes has been proposed. To set the basis for a more comprehensive delineation of their mode of action, we chemogenomically profiled the cellular effects of bis(citronellalthiosemicarbazonato)nickel(II) [Ni(S-tcitr)_2_] using the unicellular eukaryote *Saccharomyces cerevisiae* as a model organism. Two complementary genomic phenotyping screens led to the identification of 269 sensitive and 56 tolerant deletion mutant strains and of 14 genes that when overexpressed make yeast cells resistant to an otherwise lethal concentration of Ni(S-tcitr)_2_. Chromatin remodeling, cytoskeleton organization, mitochondrial function and iron metabolism were identified as lead cellular processes responsible for Ni(S-tcitr)_2_ toxicity. The latter process, and particularly glutaredoxin-mediated iron loading of RNR, was found to be affected by Ni(S-tcitr)_2_. Given the multiple pathways regulated by glutaredoxins, targeting of these proteins by Ni(S-tcitr)_2_ can negatively affect various core cellular processes that may critically contribute to Ni(S-tcitr)_2_ cytotoxicity.

## Introduction

Originally discovered as antivirals active against smallpox and other viruses^1^, thiosemicarbazones (TSC) are still attracting significant interest as anticancer agents^2–4^. The antiproliferative activity of TSCs has been initially ascribed to their metal (e.g., Fe^2+^) sequestration capacity and to the inactivation of ribonucleotide reductase (RNR), the enzyme that converts ribonucleotides into deoxyribonucleotides and whose activity correlates with cell proliferation^5,6^. However, additional targets have emerged from more recent studies, including topoisomerase II, the metalloenzymes xanthine oxidase and tyrosinase, and the mitochondria signalling pathway^7–11^.

One of the first TSC tested in phase I clinical trials was 5-hydroxy-2-formylpyridine thiosemicarbazone (5-HP)^12^. However, these trials revealed severe side effects (mainly gastrointestinal toxicity) and fast inactivation via metabolic conversion (glucuronidation) of 5-HP^4^. Further investigation has led to the development of 3-aminopyridine-2-carboxaldehyde thiosemicarbazone (3-AP, Triapine)^13^, whose efficacy is currently undergoing phase II clinical testing^14–17^. This compound shows promising activity against hematologic disorders but not solid tumours^4^. The reasons may be an inappropriate drug delivery due to a short plasma half-life, Triapine metabolic conversion, and/or rapid development of drug resistance^4^. Recently, phase I clinical trials are being performed to test Triapine in combination therapy with other anticancer drugs^18^. Since 2015^4,19,20^, phase I clinical trials were also started with new antiproliferative TSCs, namely COTI-2 and DpC, that exhibited a potent and selective activity against a variety of aggressive solid tumors in vitro and in vivo.

In addition, the TSC complexes with transition metals, such as copper and nickel, have also been shown to display interesting pharmacological properties^2,21^, including antiretroviral activity, growth inhibition of different bacteria and moulds and a tumour cell line-specific antiproliferative activity^22–29^. A multi-target mode of action also seems to apply to TSC-metal complexes, which appear to be more potent and selective antineoplastic agents than their uncomplexed counterparts. In previous studies in which we compared a series of TSCs derived from different natural compounds and chemically modified analogues, we have shown a superior bioactivity of their TSC metal-complexes compared to the corresponding metal-free ligands and pointed to bis(S-citronellalthiosemicarbazonato)-copper(II) and nickel(II) complexes as the most effective ones^25,30,31^. Because of the non-redox nature and lower biological abundance of the nickel(II) ion, the latter complex, hereafter designated as Ni(S-tcitr)_2_, was subjected to a more detailed characterization^29,32,33^. As revealed by these studies, Ni(S-tcitr)_2_ is able to cross the cell membrane and, upon internalization, it causes genotoxic stress, ultimately leading to proliferation blockage and cell death. The above effects appear to be distinct from those elicited by metal-free TSC ligands and independent from the p53 (wild-type or mutated) state, but highly selective for cycling cells such as phytoemagglutinin-stimulated lymphocytes and the human histiocytic lymphoma cell line U937^32,33^.

Despite this fairly extensive characterization, a unifying mode of action explaining the basic cellular and molecular mechanisms underlying Ni(S-tcitr)_2_ cytotoxicity is presently not available –particularly, a lack of information that not only precludes a more causal understanding of the primary targets of Ni(S-tcitr)_2_ but also a reliable and easily accessible cellular readout for its further chemical improvement.

We thus have set out to use the unicellular eukaryote *Saccharomyces cerevisiae* as a simplified, but genetically highly tractable model system for an in-depth investigation of the cellular and molecular bases of Ni(S-tcitr)_2_ cytotoxicity. In particular, we took advantage of a collection of yeast haploid strains individually deleted in all (~ 5,000) non-essential genes to perform a fitness profiling. The aim of this screening, also known as ‘Homozygous deletion profiling’ (HOP), was to identify gene products that reduce or enhance compound toxicity, and whose deletion thus causes an increase or a reduction in Ni(S-tcitr)_2_ sensitivity. As a complementary screening approach, we used ‘Multicopy suppression profiling’ (MSP), in which a wild-type strain is transformed with a collection of ORFs representative of the whole yeast proteome, in order to identify genes that when overexpressed confer an increased tolerance to Ni(S-tcitr)_2_. Combined use of these two genome-wide screenings revealed chromatin remodeling, cytoskeleton dynamics and mitochondrial function as key cellular processes negatively affected by Ni(S-tcitr)_2_. Chemogenomic profiling coupled with specific functional assays also allowed to detect an impairment of glutaredoxin-dependent iron loading onto RNR and a dysregulated iron metabolism as primary targets of Ni(S-tcitr)_2_ toxicity.

## Results

### ***Deletion mutant profiling of Ni(S-tcitr)***_***2***_*** cytotoxicity***

To identify genes and pathways involved in the response to Ni(S-tcitr)_2_, we first conducted a chemogenomic profiling using the yeast haploid deletion collection (see ‘Methods’ for details). Similar to the human macrophage cell line U937^32^*, S. cerevisiae* was also found to be sensitive to Ni(S-tcitr)_2_, with complete growth suppression at 200 µM. A sublethal concentration of 50 µM and a nearly lethal concentration of 150 µM were thus used to identify Ni(S-tcitr)_2_ sensitive and resistant mutants, respectively.

Mutant strains that proved to be sensitive or resistant in at least three out of four screens were subjected to an independent validation by serial dilution (‘spot’) assays. Sensitive mutants were classified as ‘high’ (HS), ‘medium’ (MS) and ‘low’ (LS) sensitivity according to the severity of the phenotypes displayed (Supplementary Fig. S1a; see ‘Methods’ for details). Mutants capable of growing in the presence of the highest (150 µM) Ni(S-tcitr)_2_ concentration were cumulatively classified as ‘resistant’ (Supplementary Fig. S1b).

**Figure 1:**
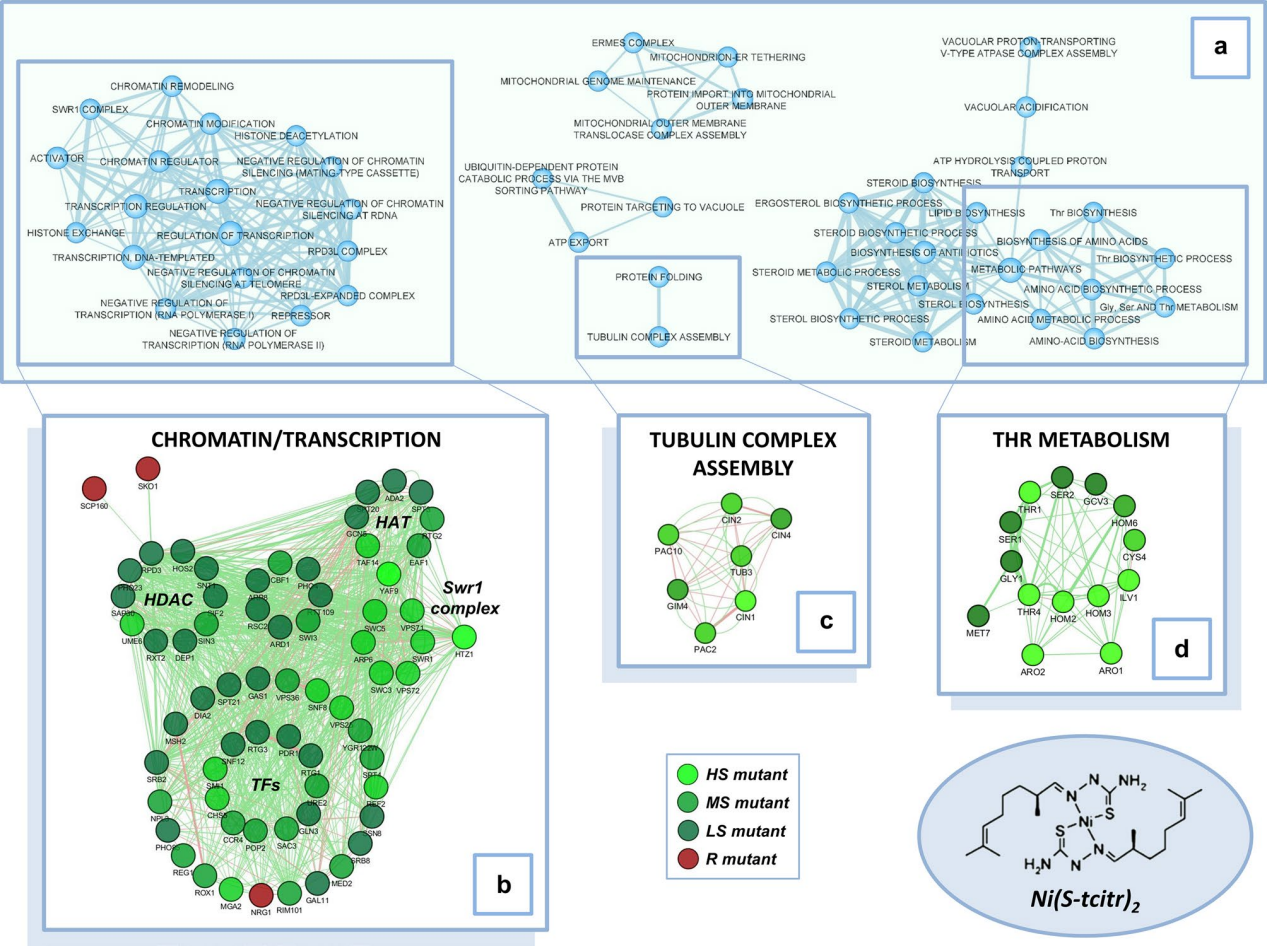
Biological pathways associated with the response to Ni(S-tcitr)_2_ in yeast. (**a**) Interaction maps of the genes that cause Ni(S-tcitr)_2_ sensitivity or resistance when deleted, generated with the functional annotation tool DAVID and visualized with the Enrichment Map plugin of Cytoscape. (**b–d**) same as (**a**) but applied to the indicated gene networks (‘chromatin/transcription’, ‘tubulin complex assembly’ and ‘Thr metabolism’ in panels b, c and d, respectively) and visualized with the Cytoscape (GeneMANIA plugin). Red and green nodes indicate genes that confer either Ni(S-tcitr)_2_ resistance or sensitivity when deleted. Each node represents a functional category (e.g., a GO term or a KEGG pathway) and the width of the edges increases with the degree of gene overlap between the two connected categories; node size increases with the number of genes annotated to each functional category.

A total of 269 sensitive and 56 resistant mutant strains were thus identified (Supplementary Table S1), nearly 50% of which have a human ortholog. As revealed by an enrichment analysis with the DAVID Functional Annotation Tool (Fig. 1), specific cellular processes appear to be targeted by Ni(S-tcitr)_2_. Among the most significant identified by Gene Ontology (GO) ‘Biological process’ analysis performed on the genes causing drug sensitivity when deleted (Table 1), there are ‘chromatin organization’ (*P*-value = 1.61E−09) and ‘regulation of gene expression’ (*P*-value = 1.72E−07). Other biological processes highly represented among the Ni(S-tcitr)_2_ sensitive mutants are ‘threonine metabolic process’ (*P*-value = 8.86E−07), ‘aromatic compound biosynthetic process’ (*P*-value = 8.15E−06) and ‘tubulin complex assembly’ (*P*-value = 5.70E−04).

**Table 1:** Biological processes associated with genes causing Ni(S-tcitr)_2_ sensitivity when deleted.

GO term^a^	*P*-value
Chromatin organization	1.61E−09
Regulation of gene expression	1.72E−07
Regulation of transcription, DNA-templated	7.08E−07
Threonine metabolic process	8.86E−07
Aromatic compound biosynthetic process	8.15E−06
Regulation of chromatin silencing at rDNA	0.00057
Tubulin complex assembly	0.00057
Histone exchange	0.00254
Vacuolar acidification	0.00363
Mitochondrion localization	0.0059

^a^Biological process Gene Ontology (GO) terms were identified and evaluated for statistical significance (*P*-value < 0.01) with the GO TermFinder program (https://www.yeastgenome.org/cgi-bin/GO/goTermFinder.pl).

**Figure 2:**
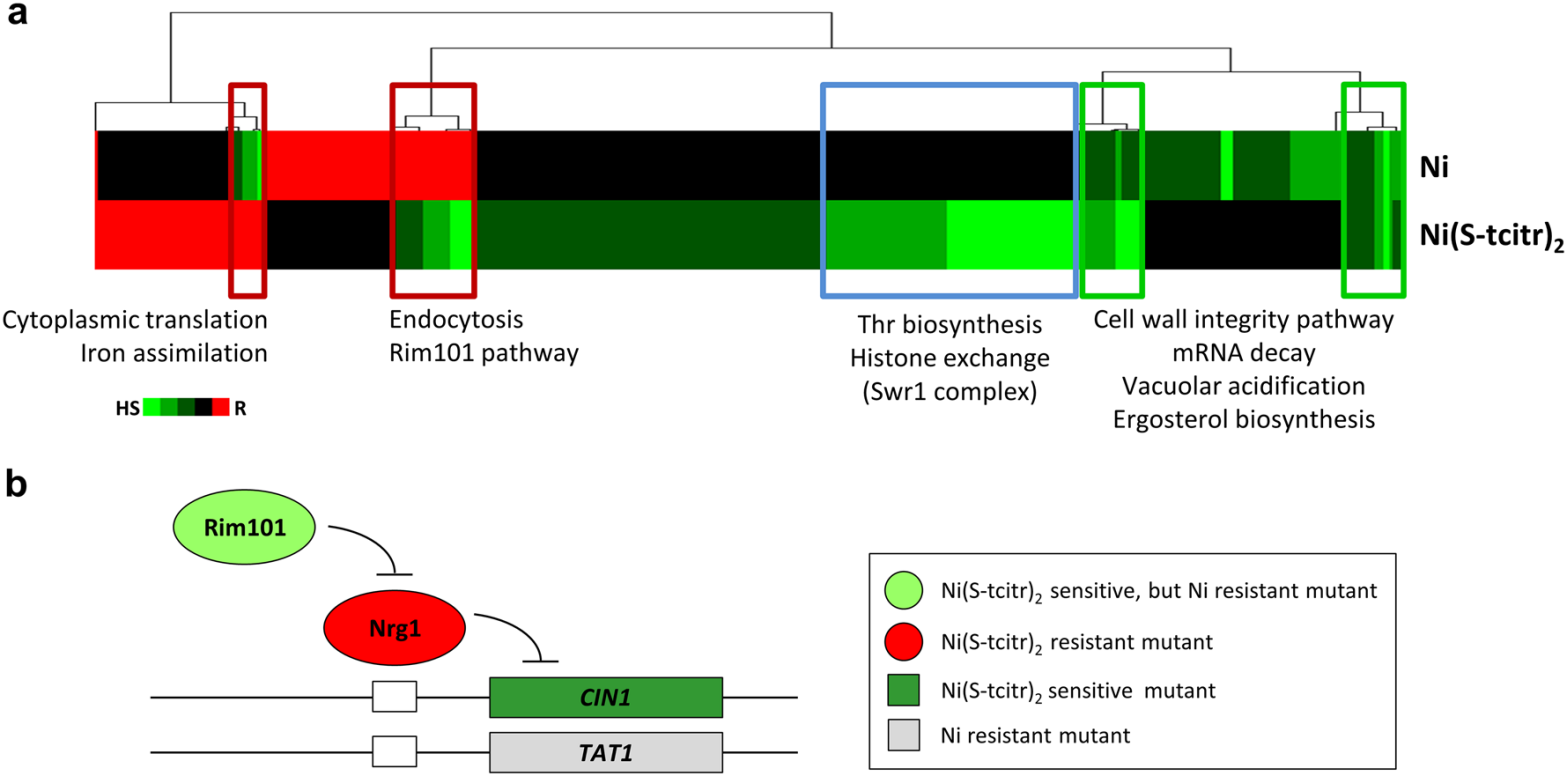
Distinct chemogenomic profiles of Ni(S-tcitr)_2_ and free nickel ions. (**a**) Two-dimensional hierarchical clustering of the chemical-genetic profiles of Ni(S-tcitr)_2_ and NiCl_2_ in yeast. Genes and compounds are represented on the vertical and horizontal axis; chemical-genetic interactions are shown in green and red for sensitive and resistant mutants, respectively. Genes involved in ‘threonine (Thr) biosynthetic process’ and ‘histone exchange (Swr1 complex)’ displayed chemical-genetic interactions only with Ni(S-tcitr)_2_. Deletion of genes involved in ‘endocytosis’, ‘Rim101 pathway’, ‘cytoplasmic translation’ and ‘iron assimilation’ displayed opposite phenotypes (sensitivity vs resistance) when treated with Ni(S-tcitr)_2_ or NiCl_2_. (**b**) Rim101 pathway deletion mutants differently respond to Ni(S-tcitr)_2_ or NiCl_2_ in *S. cerevisiae*. Data for NiCl_2_ were derived and re-elaborated from Ruotolo et al*.*^34^.

‘Cytoplasmic translation’ (*P*-value = 3.70E−04) and ‘iron assimilation’ (*P*-value = 2.17E−02) were the only significant biological processes we have identify among the resistant mutants (Fig. 2a).

To find out whether the observed phenotypes are due to nickel ions per se or to the TSC-metal complex, we compared the chemogenomic profile of Ni(S-tcitr)_2_ with that previously determined for free nickel ions^34^ (Fig. 2a). 118 mutant strains were previously found to be sensitive to a sublethal (2.5 mM) concentration of Ni^2+^^34^, compared to the 269 mutants that turned out to be sensitive to Ni(S-tcitr)_2_. Only a 15% overlap was observed between Ni(S-tcitr)_2_- and Ni^2+^-sensitive mutants and only one deletion (*sap190Δ*) conferred a tolerant phenotype to both stressors. Among the shared Ni(S-tcitr)_2_- and Ni^2+^-sensitive mutants there are strains deleted in genes coding for multiple components of general stress response pathways such as the cell wall integrity, mRNA decay, vacuolar acidification, and ergosterol biosynthesis pathways (Figs. 1a and 2a). Of note, 37 strains deleted in gene products involved in protein synthesis, endocytosis, iron assimilation and the Rim101 pathway displayed opposite responses (sensitivity vs resistance) against the two stressors (Fig. 2a).

Particularly interesting are the opposite phenotypes displayed by the Rim101 pathway mutants, which in addition to Rim101 include seven proteins involved in the proteolytic activation and/or functionality of this regulator. Mutations in this pathway, which is involved in different processes including the positive regulation of nickel uptake, make yeast cells sensitive to Ni(S-tcitr)_2_ but resistant to otherwise lethal concentrations of free Ni^2+^ ions. In fact_,_ Rim101 negatively regulates Nrg1, a repressor of the *TAT1* gene encoding a nickel transporter that appears to be causally involved in Ni^2+^ but not Ni(S-tcitr)_2_ resistance (Fig. 2b). Conversely, *NRG1* deletion makes yeast cells resistant to Ni(S-tcitr)_2_ (Supplementary Table S1), thus suggesting that one (or more) genes normally repressed by this negative regulator may be crucial for the response to the TSC-nickel complex. A particularly relevant candidate among the genes negatively regulated by Nrg1^35^ is a non-essential gene (*CIN1*) coding for a protein involved in β-tubulin folding –a process that was linked to Ni(S-tcitr)_2_ sensitivity (Table 1 and Fig. 1). In keeping with this hypothesis, *CIN1* gene deletion strongly enhanced Ni(S-tcitr)_2_ but not Ni^2+^ sensitivity^34^ (Fig. 2b).

Altogether, the above results indicate that free and TSC-complexed nickel engage distinctively different targets and detoxification pathways, and that intracellular Ni^2+^ release (if any) is absolutely negligible, at least from a biological point of view.

### Potential targets of Ni(S-tcitr)_2_ identified by multicopy suppression analysis

Multicopy suppression profiling (MSP), an approach complementary to deletion mutant profiling, was used next to identify yeast genes that suppress Ni(S-tcitr)_2_ toxicity when overexpressed and thus lend themselves as potential targets of Ni(S-tcitr)_2_. 319 MSP-positive clones, i.e., transformants tolerating an otherwise lethal concentration of Ni(S-tcitr)_2_, were isolated and further characterized (see ‘Methods’ for details). A total of 14 non-redundant single-ORF clones were thus identified (Table 2). The suppressor genes retrieved from MSP analysis encode for proteins functionally related to pathways such as chromatin remodeling (*SET4*, *RTT106*), mitochondrial function (*MGE1*, *MME1*, *TIM9*) and iron metabolism (*AFT1*; *GRX3, MGE1*), that also emerged from the HOP assay (Table 1 and Fig. 1). Other MSP-positive genes are involved in translational elongation (*TEF4*), sterol biosynthesis (*UPC2*), carbohydrate metabolism (*YMR099C*) and in the Pleiotropic Drug Resistance (PDR) network. The latter group includes Pdr5, the major plasma membrane ATP binding cassette (ABC) transporter, the transcriptional regulators Pdr1^36^ and Pdr3^37^, and Ssz1, a ribosome-associated, Hsp70-family also involved in ‘pleiotropic drug resistance’ as a positive regulator of Pdr1^38^.

**Table 2:** Multicopy suppressors of Ni(S-tcitr)_2_ sensitivity.

Gene name	Number of clones identified	Gene product function
*AFT1sh*	2	Truncated version of the transcription factor involved in iron utilization and homeostasis
*GRX3*	7	Monothiol glutaredoxin
*MGE1*	9	Mitochondrial matrix co-chaperone
*MME1*	4	Transporter of the mitochondrial inner membrane involved in magnesium export
*PDR1*	21	Transcription factor that regulates the pleiotropic drug response
*PDR3*	94	Transcriptional activator of the pleiotropic drug resistance network
*PDR5*	10	Multidrug transporter actively regulated by Pdr1p
*RTT106*	4	Histone chaperone, involved in regulation of chromatin structure in both transcribed and silenced chromosomal regions
*SET4*	11	Protein of unknown function, containing a SET domain; putative involvement in chromatin remodeling
*SSZ1*	48	Hsp70 protein involved in pleiotropic drug resistance via sequential activation of *PDR1* and *PDR5*
*TEF4*	86	Gamma subunit of translation elongation factor eEF1B
*TIM9*	5	Protein of the mitochondrial intermembrane space; forms a complex with Tim10p that delivers hydrophobic proteins to the TIM22 complex for insertion into the inner membrane
*UPC2*	9	Sterol regulatory element binding protein; induces sterol biosynthetic genes upon sterol depletion
*YMR099C*	9	Glucose-6-phosphate 1-epimerase (hexose-6-phosphate mutarotase), likely involved in carbohydrate metabolism

### Ni(S-tcitr)_2_ treatment interferes with chromatin remodeling

Chromatin remodeling emerged as a major class of HOP- and MSP-positive hits (Tables 1 and 2; Fig. 1), which included nucleosome modifiers such Swr1 and the Rpd3L and SAGA complexes, but also general transcription factors (e.g., Pdr1) and components of the Mediator complex (Supplementary Table S1 and Fig. 1a,b). Two types of experiments were performed to further characterize the involvement of chromatin modification processes in the cellular response to Ni(S-tcitr)_2_.

First, we wished to find out whether Ni(S-tcitr)_2_ affects heterochromatin silencing at telomeric ends. To this end, we used an engineered yeast strain (DG28^39^), in which the selectable *URA3* gene is inserted into the subtelomeric region of chromosome VII (left arm)—a chromosomal region that allows basal levels of transcription but is prone to chromatin compaction and heterochromatin-mediated gene silencing. This strain was exposed to the DMSO (vehicle) or to increasing concentrations of Ni(S-tcitr)_2_ and subsequently plated on medium containing the uracil precursor analogue 5-fluoro-orotic acid (5-FOA), which in the presence of a functional *URA3* gene is converted into the toxic, cell death-causing mutagen 5-fluoro-uracil (5-FU). Silencing of the sub-telomerically located *URA3* gene and lack of 5-FU production was thus monitored by measuring the number of 5-FOA-resistant (5-FOA^R^) colonies. This increased in a dose-dependent manner in the presence of increasing Ni(S-tcitr)_2_ concentrations, thus indicating the occurrence of repressive chromatin remodeling and *URA3* gene silencing as a consequence of Ni(S-tcitr)_2_ treatment (Supplementary Table S2).

**Figure 3:**
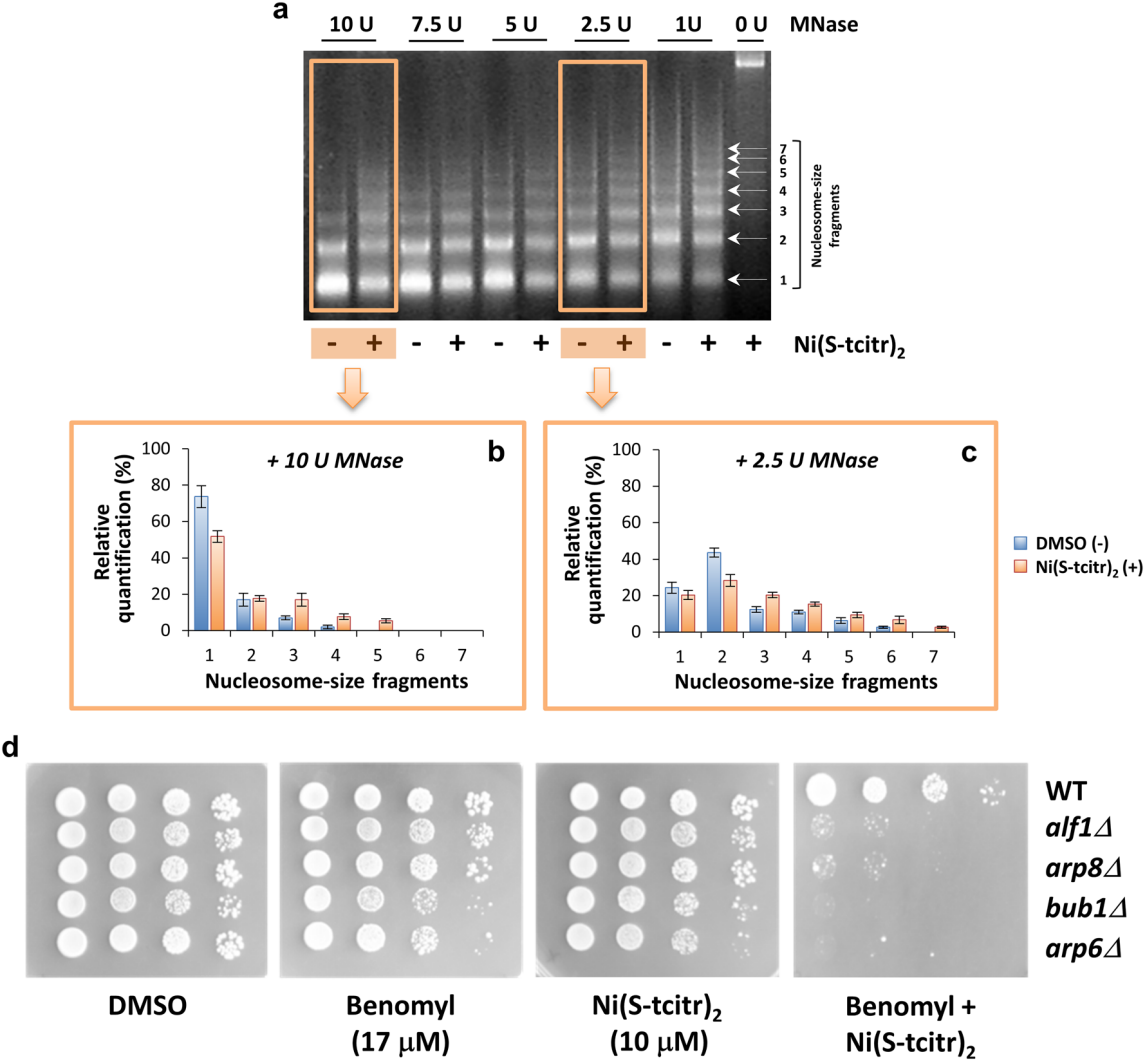
Ni(S-tcitr)_2_ affects chromatin remodeling and microtubule cytoskeleton functionality. (**a**) Effect of Ni(S-tcitr)_2_ treatment on in vivo chromatin remodeling. Equal amounts of spheroplasts from yeast cells treated with Ni(S-tcitr)_2_ or DMSO (vehicle) were subjected to chromatin digestion with micrococcal nuclease (MNase); purified DNA was then fractionated by agarose gel electrophoresis and visualized by ethidium bromide staining (see ‘Methods’ for details). A representative gel picture shows different patterns of MNase digestion products. (**b,c**) Relative quantification of nucleosome-size fragments obtained from digestion of Ni(S-tcitr)_2_-treated and control (DMSO) chromatin samples with high (10 U; **b**) or low (2.5 U; **c**) amounts of MNase. (**d**) Synergistic toxicity of Ni(S-tcitr)_2_ and the antimicrotubule drug benomyl. Ten-fold serial dilutions of wild-type (WT) cells and yeast mutant strains deleted in genes coding for chromatin remodeling (*arp6Δ* and *arp8Δ*), microtubule (*alf1Δ*) or cell cycle checkpoint components (*bub1Δ*) were plated onto YPD agar plates containing sublethal concentrations of benomyl (17 µM) and /or Ni(S-tcitr)_2_ (10 µM) as indicated, and incubated for 2 days at 28 °C.

In a second experiment, we evaluated the effect of Ni(S-tcitr)_2_ on chromatin remodeling by measuring the accessibility of micrococcal nuclease (MNase) to nucleosomal DNA. As shown in Fig. 3a, distinctively different MNase digestion patterns were observed with formaldehyde-fixed chromatin derived from vehicle- or Ni(S-tcitr)_2_-treated cells. In particular, mainly DNA fragments corresponding to mono- and di-nucleosomes were obtained from cells treated with the vehicle at the highest MNase concentration (10 U) (Fig. 3a,b). In contrast, a digestion pattern skewed toward longer DNA fragments was observed with cells treated with Ni(S-tcitr)_2_, even at the lowest (2.5 U) MNase concentrations (Fig. 3a,c). Taken together, the above results indicate that Ni(S-tcitr)_2_ can induce chromatin condensation, likely by interfering with histone (de)acetylases and other chromatin-remodeling components. These results seem to be in line with the identification of large interlaced DNA aggregates observed in the presence of Ni(S-tcitr)_2_, but not with the free metal ion, reported in our previous papers^31,33^.

### Ni(S-tcitr)_2_ treatment affects cytoskeleton organization

Deletion of genes coding for microtubule-associated and other cytoskeleton-related components also causes Ni(S-tcitr)_2_ sensitivity (Table 1 and Fig. 1a,c), an indication that the TSC-Ni^2+^ complex may interfere with cytoskeleton network dynamics. To test this hypothesis, we used serial dilution assays to evaluate the potential synergic toxicity of a combination of Ni(S-tcitr)_2_ and benomyl, an inhibitor of tubulin polymerization (Fig. 3d; see ‘Methods’ for details). To this end, wild-type yeast cells and a subset of cytoskeleton-related, Ni(S-tcitr)_2_-sensitive mutants—deleted in genes coding for an α-tubulin folding protein (*alf1Δ*), two actin-related proteins with also a role in chromatin remodeling (*arp6Δ* and *arp8Δ*) and a protein kinase involved in cell cycle checkpoint in response to spindle damage (*bub1Δ*)—were grown in the presence of sublethal concentrations of Ni(S-tcitr)_2_ and benomyl. While treatment with either compound alone did not appreciably affect viability, co-treatment with Ni(S-tcitr)_2_ and benomyl dramatically worsened the growth phenotype of the Ni(S-tcitr)_2_-sensitive, cytoskeleton-related mutants (Fig. 3d). This synergistic effect of the two compounds indicates that they target the same process and suggest that in addition to chromatin remodeling, cytoskeleton organization and microtubule functionality is an important target of Ni(S-tcitr)_2_.

### Impaired deoxyribonucleoside triphosphate synthesis in Ni(S-tcitr)_2_-treated yeast cells

Deletion of genes involved in threonine metabolism (e.g., *AAT2*; *HOM2, HOM3, HOM6*; *THR1, THR4*; *GLY1; LST4*) and tricarboxylic acid cycle regulation (*RTG1* and *RTG3*) also determines an increased sensitivity to Ni(S-tcitr)_2_ (Table 1 and Supplementary Table S1; Fig. 1d). In addition to their role in amino acid metabolism, the above pathways generate key intermediates and precursors (e.g., glycine) for de novo purine biosynthesis, thus increasing dNTP production and enabling a compensatory response to RNR deficiency or inactivation^40^. Given previous reports on RNR inhibition by TSCs^6,21^, and the fact that deletion of the gene (*RNR4*) coding for a component of the RNR small subunit causes hypersensitivity to Ni(S-tcitr)_2_ (Supplementary Table S1), we hypothesized a negative effect of Ni(S-tcitr)_2_ on this enzyme.

**Figure 4:**
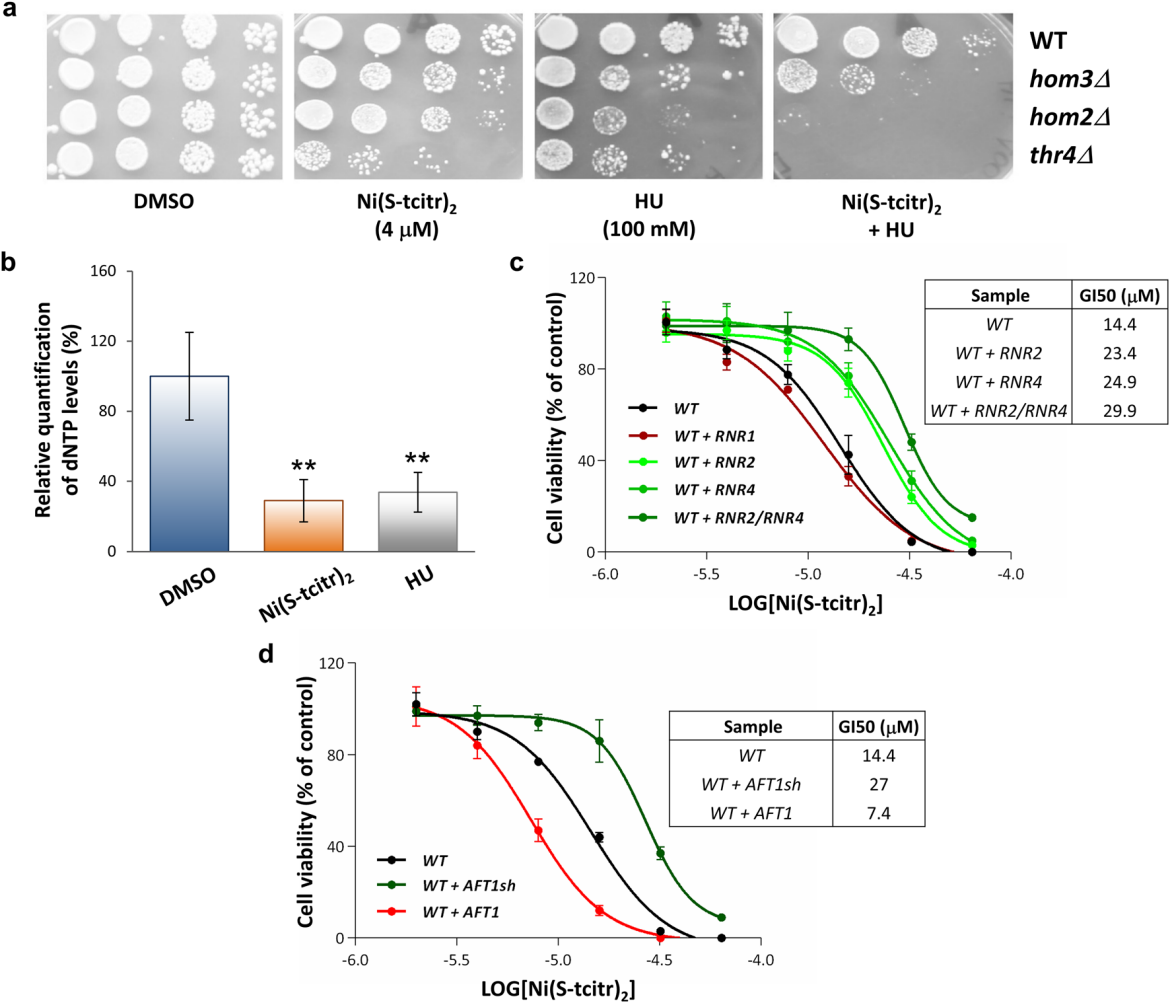
Effects of Ni(S-tcitr)_2_ on dNTP synthesis and iron trafficking pathways. (**a**) Synergistic toxicity of Ni(S-tcitr)_2_ and HU. Serial dilutions of WT yeast cells and of the indicated threonine biosynthesis deletion mutant strains were plated on YPD plates containing sublethal concentration of Ni(S-tcitr)_2_ (4 µM) and/or of the RNR inhibitor hydroxyurea (HU; 100 mM) as indicated. (**b**) Relative quantification data derived from dNTP levels measurements performed on Ni(S-tcitr)_2_-and HU-treated WT yeast cells as indicated. Data were analyzed by one-way ANOVA followed by Dunnett comparison post-hoc test (**P-value < 0.01). (**c**) Survival curves of yeast strains overexpressing specific RNR subunits. WT cells transformed with genes coding for the R1 (*RNR1*) or the R2 (*RNR2* and *RNR4*) subunits were grown in the presence of increasing concentrations of Ni(S-tcitr)_2_; cells transformed with the empty expression vectors (YEplac195 and YEplac112) served as controls. For each overexpressing strain, the results are expressed as percentage of cell viability relative to the control cells (treated with DMSO; arbitrarily set to 100%). The half maximal growth inhibition (GI50) values derived from these experiments are calculated using GraphPad Prism 6 software and shown in the inset. (**d**) Survival curves of yeast strains overexpressing the full-length (*AFT1*) and the truncated form (*AFT1sh*) of the iron regulon master regulator Aft1. WT cells transformed with genes coding for Aft1 or Aft1sh were grown in the presence of the indicated concentrations of Ni(S-tcitr)_2_; cells transformed with the empty expression vector YEplac195 served as controls. Data were analyzed as described above in the legend to panel 4c.

To test this hypothesis, we initially examined the effect of a co-supplementation of Ni(S-tcitr)_2_ and hydroxyurea (HU), a chemotherapeutic agent and a known inhibitor of RNR^41^, on the viability of a subset of the above metabolic mutants involved in threonine metabolism (*hom3Δ*, *hom2Δ* and *thr4Δ*). As shown in Fig. 4a, a synergistic effect was observed upon treatment with both Ni(S-tcitr)_2_ and HU.

Next, we evaluated the ability of Ni(S-tcitr)_2_ to interfere with RNR activity, thus leading to a reduction of cellular dNTP levels. These were determined with an indirect assay measuring the ability of cell-free extracts derived from Ni(S-tcitr)_2_-, HU- or DMSO-treated yeast cells to support in vitro DNA synthesis^42–44^ (see ‘Methods’ for details). As shown in Fig. 4b, a decreased DNA synthesis compared to the DMSO control was observed in extracts from yeast cells treated with HU but also with Ni(S-tcitr)_2_, indicating a reduction of dNTP levels induced by both compounds and likely due to RNR inhibition.

We followed-up to this result by testing tolerance to Ni(S-tcitr)_2_ of yeast cells overexpressing individual RNR subunits. *S. cerevisiae* RNR is a tetramer composed by a homodimer of the large catalytic subunit R1 (Rnr1) and a small subunit R2 composed of an Rnr2-Rnr4 heterodimer^45^. Overexpression of *RNR2* and *RNR4*, but not *RNR1*, relieved Ni(S-tcitr)_2_ cytotoxicity, with a more than additive effect when both subunits were co-overexpressed, compared to single-subunit overexpressors (Fig. 4c). The Rnr2 subunit contains a diferric tyrosyl radical cofactor [Fe(III)_2_-Tyr•] that is essential for RNR activity and represents the main target of HU. The Rnr4 subunit, instead, is not directly involved in tyrosyl radical formation, but promotes the assembly of the Rnr2-Rrn4 heterodimer and is required for iron loading onto Rnr2 through a glutaredoxin (Grx3/Grx4)- and glutathione (GSH)-dependent mechanism^46,47^. Interestingly, *GRX3* was retrieved as a multicopy suppressor of Ni(S-tcitr)_2_ toxicity (Table 2), whereas yeast strains defective in GSH biosynthesis (*gsh1Δ* and *gsh2Δ*) were identified as Ni(S-tcitr)_2_-sensitive mutants (Supplementary Table S1).

At variance with direct R2 targeting by HU, an indirect RNR inhibition mechanism, centered on interference by Ni(S-tcitr)_2_ with glutaredoxin-mediated iron loading onto the R2 subunit, can thus be envisaged. In keeping with this hypothesis, genetic evidence indicates that Ni(S-tcitr)_2,_ but not HU^48^, also interferes with glutaredoxin-dependent iron sensing mediated by Aft1^49^, the master regulator of the iron regulon^50^. Defective iron sensing by this regulator is known to induce persistent activation of genes involved in iron uptake even under iron-sufficient conditions^51^. Indeed, a non-functional Aft1 derivative (‘Aft1sh’) lacking the C-terminal *trans*-acting domain but capable of competing with full-length Aft1 for DNA binding, was identified as a multicopy suppressor of Ni(S-tcitr)_2_ toxicity (Table 2). Furthermore, as shown in Fig. 4d, an increased Ni(S-tcitr)_2_ tolerance was observed in yeast cells overexpressing the non-functional truncated (AFT1sh; aa. 1-487) but not the full-length functional form (aa. 1-690) of Aft1, whose expression rather confers sensitivity to Ni(S-tcitr)_2_ treatment. Of note, a similar enhancement of Ni(S-tcitr)_2_ tolerance (but an increase in HU sensitivity^48^) was observed in deletion mutants lacking the Aft1-regulated genes *FET3* and *FTR1* encoding the structural components of the high affinity iron transport system (Supplementary Table S1 and Fig. 2a).

### Ni(S-tcitr)_2_ sensitivity correlates with RNR expression levels in human cancer cell lines

**Figure 5:**
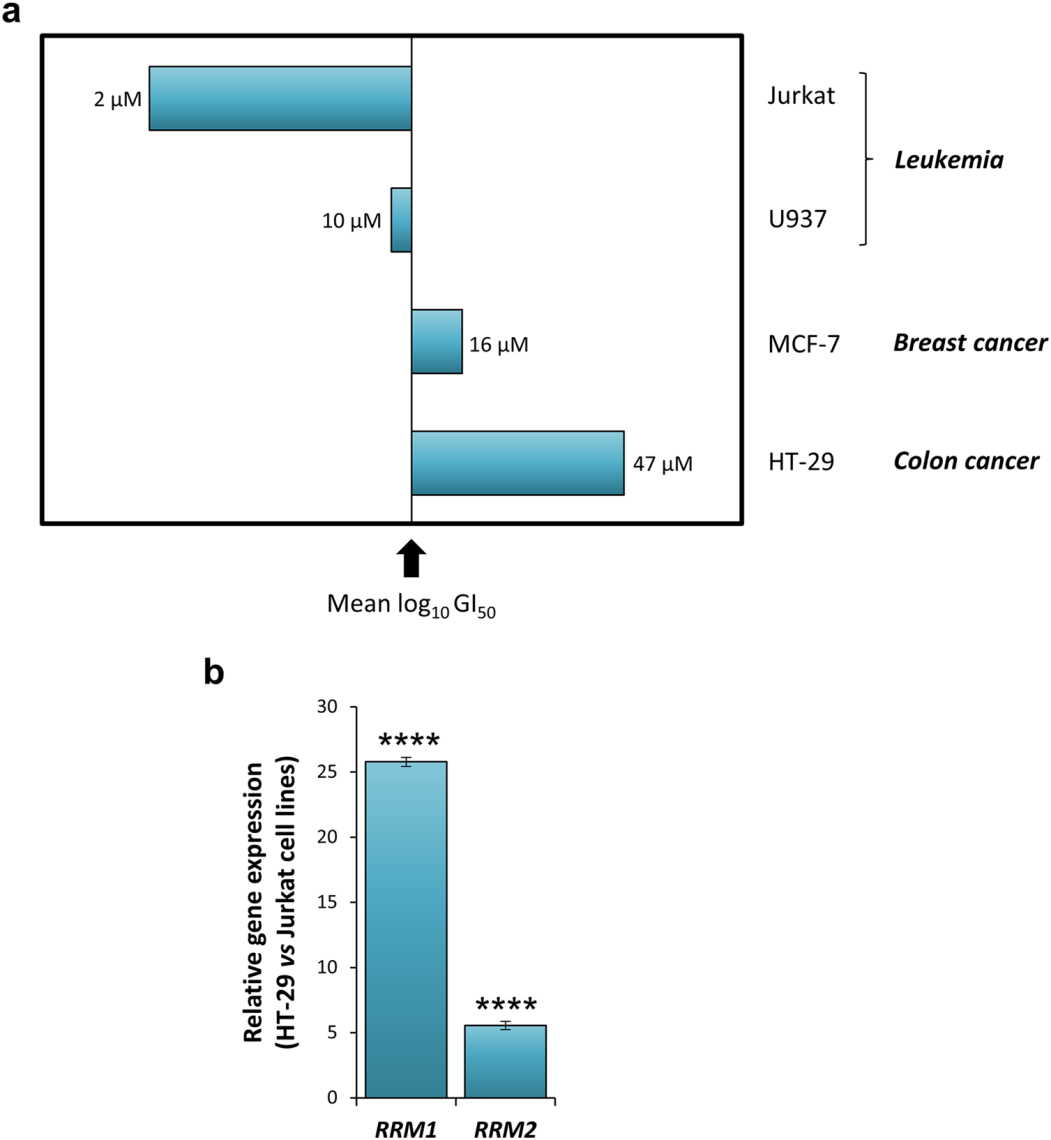
Ni(S-tcitr)_2_ sensitivity of human cancer cell lines correlates with the transcriptional levels of RNR subunits. (**a**) Antiproliferative activity of Ni(S-tcitr)_2_ on the indicated human cancer cell lines. GI_50_ values, determined after a 24 h treatment, are indicated. The midline in the graph represents the mean of the log_10_ GI_50_ values measured across the whole set of the human cancer cell lines comprised within the ‘US National Cancer Institute 60 human tumor cell line anticancer drug screen’^52^; this mean GI_50_ value was subtracted from those determined for each individual cell line. Hypersensitive and hyposensitive cell lines (compared to the mean sensitivity of the whole set of analyzed cell lines) are shown as left-deflecting and right-deflecting bars, respectively. (**b**) Relative abundance of the mRNAs coding for the *RRM1* and *RRM2* subunits of human RNR in the least Ni(S-tcitr)_2_ sensitive (HT-29) and the most sensitive (Jurkat) cancer cell line. Transcript levels were determined by Real-Time RT-PCR after 24 h growth under standard conditions; data, which represent the average ± SD of three biological replicates, were normalized using *GAPDH* as a housekeeping reference gene. Data were analyzed by one-way ANOVA followed by Dunnett comparison post-hoc test (****P-value < 0.0001).

Ni(S-tcitr)_2_ has previously been shown to inhibit proliferation of the histiocytic lymphoma, human cancer cell line U937 through a cytotoxic mechanism involving apoptosis^32^. To begin to assess the functional overlap between the molecular responses to Ni(S-tcitr)_2_ identified in *S. cerevisiae*—particularly, the observed impairment of dNTP biosynthesis and the R2/R4 subunit-dependent negative effect on RNR—we performed a growth-inhibition screening on the different cell lines comprised within the ‘US National Cancer Institute 60 human tumor cell line anticancer drug screen’^52^ (see ‘Methods’ for details)_._ As shown in Fig. 5a, which reports the results (expressed as 50% growth inhibition; GI_50_) of the two most sensitive and the two least sensitive cell lines, the strongest effects on cell proliferation were observed in human cell lines derived from hematologic malignancies. In particular, the highest sensitivity to Ni(S-tcitr)_2_ (GI_50_ = 2 µM) was observed with the immortalized T-lymphocyte, Jurkat cell line, whereas the colon adenocarcinoma HT-29 cell line displayed the lowest sensitivity (GI_50_ = 47 µM). Interestingly, evaluation of RNR expression levels in the above cell lines revealed an inverse relationship between the amount of RNR transcripts and Ni(S-tcitr)_2_ sensitivity (Fig. 5b). In fact, the relative abundance of the R1 (*RRM1*) but also of the R2 (*RRM2*) subunit were 25-fold and fivefold higher in the poorly sensitive HT-29 compared to the highly sensitive Jurkat cell line. As observed for other anticancer compounds that directly (or indirectly) interfere with RNR activity^53–56^, these data suggest a buffering effect of RNR levels on Ni(S-tcitr)_2_ cytotoxicity, leading to an increased tolerance to this organometallic compound in the presence of higher cellular concentrations of the RNR target.

## Discussion

The aim of this study was to gain genome-wide insight on the target of the antiproliferative TSC-nickel complex Ni(S-tcitr)_2_ using genetic tools available in the model eukaryote *S. cerevisiae.*

The data presented in this work support a fairly peculiar mechanism for Ni(S-tcitr)_2_ cytotoxicity, centered on the inhibition of monothiol Grx activity, which appears to be distinct from the mode of action of metal-free TSCs and other related compounds^4^. Grxs are known to regulate iron cellular trafficking in yeast and human cells by promoting the GSH-dependent transfer of Fe^2+^ from a cytosolic ‘labile iron pool’ to iron-dependent enzymes (including RNR) and organelles, especially mitochondria^49,51,57,58^. In line with these observations, we have found that the overexpression of *GRX3* increased tolerance to Ni(S-tcitr)_2_, whereas deletion of GSH biosynthetic genes caused sensitivity to Ni(S-tcitr)_2_ (Table 2 and Supplementary Table S1). Yeast Grxs, in particular, contain an unusual GSH-ligated Fe–S cluster that is absolutely required for iron loading onto the R2 subunit of RNR, which, in turn, is essential for enzyme activity^49^. RNR appears to be a particularly relevant potential target of Ni(S-tcitr)_2_, as indicated by the reduction of the intracellular dNTP pool caused by Ni(S-tcitr)_2_ treatment, and by the increased tolerance to Ni(S-tcitr)_2_ associated with overexpression of the iron-containing R2 subunits (Rnr2 and Rnr4), but not of the catalytic R1 subunit (Fig. 4). Also, in line with interference of Ni(S-tcitr)_2_ with RNR activity, is the inverse relationship between RNR expression levels and Ni(S-tcitr)_2_ sensitivity observed in human cell lines (Fig. 5).

Grx activity is also essential for the nuclear export of the transcription factor Aft1 in response to iron sufficiency and, conversely, a depletion of Grxs causes a constitutive activation of the iron regulon mediated by Aft1^49,51^. It is thus conceivable to imagine that Ni(S-tcitr)_2_-mediated impairment of Grxs can inhibit multiple iron-dependent enzymes and pathways, with the concomitant generation of a false Fe^2+^ deficiency signal and the upregulation of the iron regulon. In fact, the results of our integrated (HOP and MSP) chemogenomic analysis point to an inverse relationship between Ni(S-tcitr)_2_ toxicity and the impairment of iron homeostasis. Specifically, we found that deletion of genes involved in Fe^2+^ uptake (e.g., the high-affinity iron transporter complex Fet3-Ftr1) as well as the production of a functionally defective form of Aft1 cause a reduction of Ni(S-tcitr)_2_ cytotoxicity (Supplementary Table S1 and Fig. 4).

The concentration of cytosolic iron have to be tightly regulated in yeast to assure a continuous supply of iron to the cell, but simultaneously to prevent its toxicity because Fe^2+^ can participate in the Fenton and Fenton-like reactions to generate deleterious reactive oxygen species (ROS). To prevent damage to cellular constituents, much of the free intracellular iron is imported into vacuoles^59^. In HOP and MSP assays, we did not observe an activation of the antioxidant stress response upon Ni(S-tcitr)_2_ treatment (Tables 1 and 2), but we found that deletion mutant strains with impaired vacuolar functionality displayed sensitivity to Ni(S-tcitr)_2_ (Fig. 1a and Table 1). Moreover, mutant cells lacking components of the ESCRT complex-dependent multivesicular body (MVB) sorting pathway were sensitive to Ni(S-tcitr)_2_ treatment (Fig. 1a and Supplementary Table S1). Through this pathway, high-affinity iron transporter Fet3-Ftr1 can be endocytosed and delivered to the vacuole for degradation, a mechanism that can be used to decrease the cytosolic iron concentration and prevent iron toxicity via the ROS generation^60^.

We have also found that Ni(S-tcitr)_2_ cytotoxicity does not appear to be associated with nickel release (which could favour the formation of an iron-TSC complex with redox activity^4^) because Ni(S-tcitr)_2_ is characterized by a chemogenomic profile distinct from that of free Ni^2+^ ions (Fig. 2). Therefore, the data presented in this paper don't seem to support ROS generation induced by Ni(S-tcitr)_2_ treatment in yeast, but favour a complex and multi-faceted mode of action based on an interference with the cellular iron homeostasis that affect diverse biological pathways, as evidenced in more recent studies^4,61^.

A defective activity of Grxs caused by Ni(S-tcitr)_2_ may also interfere with the import of iron from the cytosol into the mitochondria and with Fe–S cluster biogenesis^47,49,51^. In line with this hypothesis, deletion mutants lacking genes coding for transporters involved in mitochondrial iron trafficking such as *ggc1Δ*, previously shown to be involved in mitochondrial iron overload^62^, or *mrs4Δ*^63^, deleted in a mitochondrial high-affinity iron transporter, displayed Ni(S-tcitr)_2_ tolerance and sensitivity, respectively. Furthermore, as revealed by the cumulative results of HOP and MSP assays, multiple mitochondrial components (Supplementary Table S1 and Table 2) are needed to counteract Ni(S-tcitr)_2_ toxicity, also under fermentative growth conditions. For example, *MGE1*, one of the multicopy suppressors identified in the present study (Table 2), codes for a mitochondrial Fe–S cluster folding component. Disruption of mitochondrial Fe–S cluster biogenesis has been shown recently to affect not only mitochondrial metabolism (e.g., OXPHOS and TCA cycle), but also to interfere with other essential cellular processes, including histone and α-tubulin acetylation, through the depletion of key metabolic cofactors and intermediates such as acetyl-coA and succinate^64^. This metabolic link may explain the presence of ‘chromatin remodeling’ and ‘cytoskeleton organization’ among the predominant categories of genes that affect Ni(S-tcitr)_2_ toxicity when deleted or overexpressed.

In conclusion, this work provides a novel mechanistic framework for Ni(S-tcitr)_2_ toxicity that will form the basis for future in-depth investigations of its translational potential to human cells, especially hypersensitive cancer cells such as the Jurkat cell line, in addition to a number of easily screenable functional biomarkers for the bioactivity evaluation and further improvement of TSC-transition metal complexes.

## Methods

### Yeast strains and culture conditions

The haploid knockout collection^65^ was purchased from Open Biosystems and converted into a 384-well plate format by manual multipinning^34^. In this collection, individual ORFs were deleted in the BY4742 background (*MATα his3Δ1 leu2Δ0 lys2Δ0 ura3Δ0*), except for 79 strains for which the BY4739 parental background (*MATα leu2Δ0 lys2Δ0 ura3Δ0*) was employed.

The BY4743 diploid strain (*MATa/α his3Δ1/his3Δ1 leu2Δ0/leu2Δ0 LYS2/lys2Δ0 met15Δ0/MET15 ura3Δ0/ura3Δ0*) was used for the MSP assay.

The DG28 strain (kindly provided by Dr. Gottschling) was used for telomere silencing experiments.

Cells were grown at 28 °C in minimal synthetic (SD) medium containing 0.67% (w/v) yeast nitrogen base, the required amino acids (or bases) and 2% (w/v) glucose as a carbon source or in Yeast Extract-Peptone-Dextrose [YPD; 0.5% (w/v) yeast extract, 1% (w/v) peptone, 2% (w/v) glucose] medium. Due to the low solubility of Ni(S-tcitr)_2_ in SD medium, the screening was performed in YPD medium. For 5-FOA experiments, SD medium was supplemented with uracil (50 mg/L) and 5-FOA (1 g/L).

### Cell lines and culture conditions

The following human cancer cell lines were used in this study: HT29 (colorectal adenocarcinoma), Jurkat (acute T cell leukemia), MCF7 (breast adenocarcinoma) and U937 (histiocytic lymphoma). All cell lines were obtained from the American Type Culture Collection (ATCC). Jurkat and U937 cells were cultured in RPMI-1640, whereas HT29 and MCF7 cells in Dulbecco's Modified Eagle Medium (DMEM). All media were supplemented with 10% (v/v) fetal bovine serum, penicillin (100 U/mL), streptomycin (100 μg/mL) and L-glutamine (2 mM). Adherent cells were grown as a subconfluent monolayer. Flasks and plates were maintained at 37 °C and 5% CO_2_ in a humidified atmosphere.

### Homozygous deletion profiling and cytotoxicity assays

The screening of the yeast knockout collection was performed manually as described previously^34^. Four biological replicates were performed for each experimental condition by manually pinning ordered sets of mutants onto YPD agar plates, followed by colony size analysis after 72 h of incubation at 28 °C.

Mutant strains identified as positive (sensitive or resistant) in the primary screen were grown on YPD medium and validated using serial dilution (‘spot’) assays^66^ in which the wild-type strain was used as control (Supplementary Fig. S1). For the spot assay, mutant strains of interest were precultured in YPD medium for 24 h at 28 °C and the optical density at 600 nm (OD_600_) of individual cultures was determined; each culture was then adjusted at a concentration of 10^7^ cells/mL and serially diluted in tenfold increments. Aliquots (4 μl) of each dilution were spotted onto YPD agar plates in the presence or absence of Ni(S-tcitr)_2_ and cell growth was examined after incubation at 28 °C for 3 days. Mutant strains exhibiting a reduction in growth at the first, second or third, or fourth dilution were classified as having a HS, MS, or LS phenotype, respectively.

For GI_50_ determination, yeast cells were routinely inoculated at a final density of 5 × 10^5^ cells/mL in YPD supplemented with Ni(S-tcitr)_2_ (0–512 µM) or DMSO (vehicle). After 24 h at 28 °C, cell density was measured spectrophotometrically at 600 nm in order to determine GI_50_ values, defined as the Ni(S-tcitr)_2_ concentration causing 50% inhibition of yeast cell growth compared to untreated controls.

### Multicopy suppression profiling

The optimal Ni(S-tcitr)_2_ concentration for MSP (60 µM) was determined by culturing the diploid BY4743 strain (~ 2 × 10^5^ cells) for 48 h on SD medium, followed by replica culture in YPD medium supplemented with different concentrations (10–100 µM) of Ni(S-tcitr)_2_.

For MSP, the BY4743 strain was transformed with 4 µg of yeast genomic libraries constructed in the multicopy pSEY8 or YEp24 vectors (*URA3* selectable marker) using the lithium acetate procedure^67^, which yielded approximately 1.4 × 10^6^ transformants upon growth on SD (without uracil) agar plates at 28 °C. After 48 h, yeast colonies were replicated in YPD medium containing 60 µM Ni(S-tcitr)_2_. After an additional 24 h, colonies were picked and transferred to YPD plates containing 100 µM Ni(S-tcitr)_2_ to evaluate the tolerant phenotype of the transformants.

Plasmid DNA was extracted from each resistant clone as described by Hoffman et al*.*^68^ and used to transform DH10B *Escherichia coli* cells. Yeast plasmids were then extracted from three colonies of each *E. coli* transformant and digested with *Eco*RI and *Hind*III. This double digestion allows to distinguish empty vectors (pSEY8 or Yep24) from insert-containing vectors and to obtain the digestion pattern of the insert. Based on digestion patterns, clones were classified into several groups, and for each group, one plasmid was used to re-transform BY4743 cells. Secondary transformants were then spotted on media containing 100 µM Ni(S-tcitr)_2_ to confirm their ‘resistant’ phenotypes compared to empty vector control transformants. To identify the genes responsible for Ni(S-tcitr)_2_ toxicity mitigation, plasmid DNA extracted from resistant clones was sequenced using M13Uni and M13Rev, and TE1Fw and TET1Rv primers for pSEY8 and YEp24, respectively, and internal primers for the insert (Supplementary Table S3). When more than one gene was present in the plasmid (Supplementary Table S4), candidate genes were subcloned into the pSEY8 and/or YEplac195 vectors^69^. If no restriction site was available, candidate genes were PCR-amplified using primers complementary to constant upstream and downstream regions and subcloned into the pSEY8 vector; oligonucleotides utilized as PCR primers are listed in Supplementary Table S3. To further validate the resistant phenotypes associated with genes retrieved the genomic DNA library, plasmids were also transformed into the BY4742 strain.

Other genes not identified as multicopy suppressors but analyzed in this study (*AFT1*, *AFT1sh*, *RNR2*, *RNR4*) were PCR-amplified with their own promoters and terminators and cloned into the YEplac195 or YEplac181 vectors, as indicated in Supplementary Table S3. The *RNR1* gene was extracted from the pWJ841 plasmid^70^ and subcloned into the YEplac195 vector.

### 5-FOA-resistant colony selection

Strain DG28^39^ was pre-grown for 48 h on solid SD medium lacking uracil in order to stimulate *URA3* expression. Approximately 2.5 × 10^5^ cells/mL were then inoculated into YPD medium containing Ni(S-tcitr)_2_ (1–3 µM) or the vehicle. After 40 h at 28 °C, 2000 cells from each culture were plated on SD medium supplemented by 5-FOA (1 g/L) and the percentage of 5-FOA^R^ colonies was determined after 4 days of culture at 28 °C. In parallel, 400 cells from each culture were plated on YPD (without 5-FOA) in order to determine the total number of colony forming unit (CFU).

### Micrococcal nuclease assay

The MNase assay was performed according to Infante et al*.*^71^ starting from BY4742 cells cultured in Ni(S-tcitr)_2_ (15 µM)- or vehicle-containing YPD up to a density of 6–7 × 10^6^ cells/mL. After crosslinking with 1% (v/v) formaldehyde and spheroplast production by zymolyase treatment, DNA was digested with 1–10 units of MNase (New England Biolabs) for 30 min at 37 °C. The experiments were performed in triplicate from independent cultures.

### RNR activity assay

Yeast cells (~ 2 × 10^8^ cells) were grown exponentially at 28 °C for 4 h in YPD media supplemented with Ni(S-tcitr)_2_ (16 µM), hydroxyurea (300 mM) or vehicle before harvesting. dNTP extracts were prepared with a modified methanol extraction and boiling method^43,72^. Briefly, harvested cells were resuspended in 60% cold methanol, vortexed (10 times, 30 s each) during a 2 h incubation on ice and placed at − 20 °C overnight. The resulting extracts were then boiled for 2 min, clarified by centrifugation (15 min at 15,000 rpm) and dried in a SpeedVac Concentrator (Martin Christ Freeze Dryers). Dried material was resuspended in ice-cold water and assayed with the indirect enzymatic method based^42–44^ for its ability to support in vitro DNA synthesis primed by Dy-682 5′-modified, fluorescent oligonucleotides (Eurofins Genomics; Supplementary Table S3). The reaction mixture was assembled in a final volume of 25 µL filled-up with colorless GoTaq reaction buffer, containing 0.6 U of GoTaq DNA Polymerase (Promega), 0.2 mM dNTP mixture (without thymidine or cytidine), 0.5 µM 5′-Dy-682 oligonucleotides, 200 pg of the pYes2 vector (Thermo Fisher Scientific) as DNA template, and 5 µL of cellular dNTP extracts derived from the different treatments. DNA synthesis was performed under standard PCR conditions (35 cycles) and the resulting products were fractionated by electrophoresis on 8% polyacrylamide gels, followed by visualization and quantification with an Odyssey imaging system (LI-COR Biosciences).

### Cytotoxicity assays in human cell lines

Cells were seeded (1.5 × 10^4^ cells/mL) in a 96-well plate format (100 µL) for 24 h and treated with increasing concentrations of Ni(S-tcitr)_2_ (0.5–100 µM) for additional 24 h. Cell viability was determined by Cell-Titer 96 AQueous One Solution Cell Proliferation Assay (MTS) kit (Promega), following the manufacturer's recommendations. After treatment, MTS reagent was added to each well and the absorbance of the formazan product was measured at 450 nm after 4 h using a microplate reader (Thermo Fisher Scientific). Cytotoxicity was expressed as GI_50_ value (drug concentration inducing a 50% of maximal inhibition of cell proliferation). Viability was also evaluated by trypan blue exclusion method^73^.

### Real time PCR

2 × 10^6^ cells were seeded in flasks with complete medium. After 24 h, cells were collected and total RNA was extracted using GeneJET RNA Purification Kit (Thermo Fisher Scientific) according to manufacturer's protocol. Total RNA (1 µg) was reverse-transcribed using PrimeScript First Strand cDNA Synthesis Kit (Takara Biomedicals) according to manufacturer's protocol. After cDNA synthesis, RT-PCR was carried out using DyNAmo SYBR Green qPCR Kit (Thermo Fisher Scientific) with DNA Engine Opticon System (Bio-Rad). The comparative C_T_ method was used for relative mRNA quantification; glyceraldehyde 3-phosphate dehydrogenase gene (*GAPDH*; Suppl. Table S3 for primer details) was used for expression level normalization. Each experimental condition was performed with three biological replicates.

### Data analysis

All experiments were performed at least in triplicate, from independent clones. Data were represented as means ± standard deviation (SD). As indicated in figure legends, statistical analyses were performed by one-way ANOVA followed by Dunnett comparison post-hoc test (**, *P-*value < 0.01; ****, *P-*value < 0.0001) using the GraphPad Prism 6 software.

Gene Ontology (GO) ‘Biological processes’ associated with Ni(S-tcitr)_2_ toxicity modulating genes were identified and evaluated for statistical significance (*P*-value ≤ 1E-02) with the GO Term Finder program (https://www.yeastgenome.org/cgi-bin/GO/goTermFinder.pl). The EnrichmentMap^74^ and GeneMANIA^75^ plugins were used for network visualization of the results obtained from an analysis conducted with the DAVID Functional Annotation Tool^76^ using the Cytoscape network visualization software^77^.

Hierarchical clustering of chemogenomic data was performed with the Cluster v3.0 software^78^ and visualized using Java TreeView^79^.

## Supplementary information

Supplementary file1 (PDF 53 kb)

Supplementary file2 (XLS 147 kb)
