# Supplementary material for: Mechanistic insights on the mode of action of an antiproliferative thiosemicarbazone-nickel complex revealed by an integrated chemogenomic profiling study
Source: Sci Rep. 2020 Jun 29;10:10524. doi: 10.1038/s41598-020-67439-y (PMC7324377; doi:10.1038/s41598-020-67439-y)
Supplement: Supplementary file 1 — Supplementary file1 (PDF 53 kb) [file 41598_2020_67439_MOESM1_ESM.pdf]

Supplementary Information

Mechanistic insights on the mode of action of an antiproliferative thiosemicarbazone-nickel complex revealed by an integrated chemogenomic profiling study

Enrico Baruffini, Roberta Ruotolo, Franco Bisceglie, Serena Montalbano, Simone Ottonello, Giorgio Pelosi, Annamaria Buschini, Tiziana Lodi

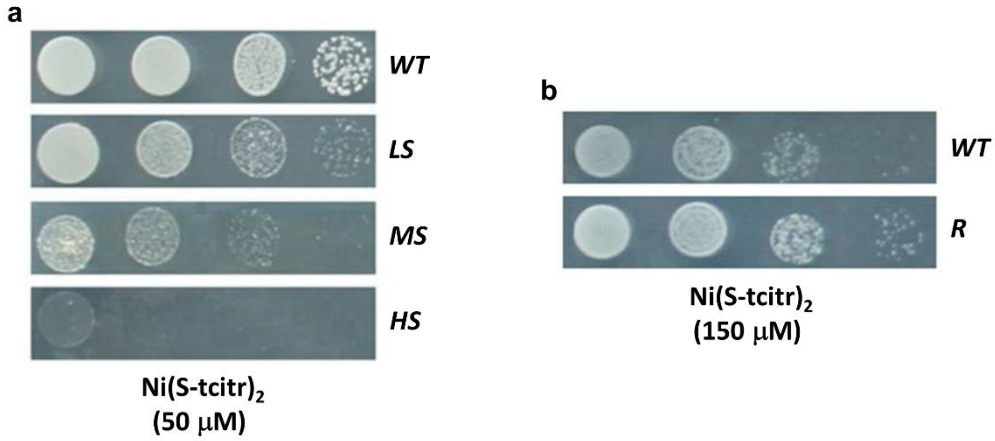

Supplementary Figure S1. Representative mutant strains displaying Ni(S-tcitr)$_2$ sensitivity (a) or resistance (b). Ten-fold serial dilutions of each yeast culture (starting from 4x10^4 cells/mL) were spotted on YPD agar plates supplemented with Ni(S-tcitr)$_2$ (50 µM or 150 µM for the identification of sensitive or resistant mutant strains, respectively). Mutant strains exhibiting various levels of drug sensitivity [high sensitivity (HS), medium sensitivity (MS), and low sensitivity (LS)] and a single class of Ni(S-tcitr)$_2$-resistant mutant strains (R) were identified.
